# DNA polymerase I is required for the Hachiman-family anti-phage defense system

**DOI:** 10.1128/jb.00140-26

**Published:** 2026-09-14

**Authors:** Saya Takita, Reoto Moroi, Haruka Terasaki, Yunosuke Shintani, Ayane Uta, Kotaro Chihara, Kotaro Kiga, Yuichi Otsuka

**Affiliations:** 1 Department of Biochemistry and Molecular Biology, Graduate School of Science and Engineering, Saitama University 13032, Saitama, Japan; 2 Department of Drug Development, National Institute of Infectious Diseases, Japan Institute for Health Security 13511, Tokyo, Japan; University of California San Francisco, San Francisco, California, USA

**Keywords:** *Escherichia coli*, bacteriophages, anti-phage defense, Hachiman, AbpA-AbpB, DNA polymerase I, RNase HI

## Abstract

Abortive infection (Abi) systems protect bacterial populations from bacteriophages by triggering growth arrest or cell death in infected cells; however, how Abi effectors are coupled to changes in host DNA replication and repair remains unclear. The widely conserved Hachiman defense system encodes a nuclease (HamA) and a helicase (HamB) that assemble into the HamAB effector complex, and *Escherichia coli* carries a related variant, AbpAB. Here, we show that DNA polymerase I (PolA) is required for AbpAB- and HamAB-mediated phage restriction, whereas RNase HI prevents AbpAB-dependent growth inhibition during uninfected growth. In a temperature-sensitive *polA* mutant (*polA12*), AbpAB- and HamAB-mediated restriction of multiple phages was lost, as was AbpAB-dependent growth arrest triggered by mitomycin C (MMC)-induced DNA damage or by expression of the T4 single-stranded DNA-binding protein gp32. Truncation analyses suggest that the PolA requirement for AbpAB-dependent growth arrest may involve the PolA-specific N-terminal 5′–3′ exonuclease domain, whereas SOS-induced polymerases II, IV, and V were not required. Pull-down assays detected PolA in association with the AbpAB complex *in vivo*; this co-detection persisted after DNase treatment, increased after MMC treatment, and decreased during T4 infection. In contrast, deletion of *rnhA*, which encodes RNase HI, rendered AbpAB expression toxic even in the absence of phage. Together, these results identify PolA and RNase HI as host factors that respectively enable Hachiman-family phage defense and prevent AbpAB-dependent growth inhibition during uninfected growth.

**IMPORTANCE:**

Abortive infection (Abi) systems can stop phage replication by forcing infected cells to arrest growth or die, but this strategy is risky if it is triggered in the absence of phage. Here, we show that two Hachiman-family Abi systems—AbpAB and HamAB—require *Escherichia coli* DNA polymerase I (PolA) to block phage growth. PolA is also required for AbpAB-dependent growth arrest triggered by DNA damage (mitomycin C) or by expression of the T4 single-stranded DNA-binding protein gp32. In contrast, deleting *rnhA* (encoding RNase HI) makes AbpAB expression toxic even in the absence of phage. These results show that host replication and repair enzymes can both support anti-phage defense and prevent Abi-associated growth inhibition during uninfected growth.

## INTRODUCTION

Bacteria have evolved diverse defense strategies to counter bacteriophage (phage) infection, which threatens their survival and proliferation (1, 2). Among them, abortive infection (Abi) is a strategy wherein infected cells undergo growth arrest or cell death, terminating phage replication prematurely and thereby protecting neighboring clonal cells (1, 3–6). In recent years, the molecular mechanisms by which many Abi effectors (toxic factors) inhibit host growth have been increasingly elucidated. In parallel, recent studies have expanded our understanding of infection-associated signals that activate Abi effectors and the upstream regulatory mechanisms governing their activation.

The Hachiman system is a widely distributed Abi defense module that functions as a complex (HamAB) composed of a nuclease (HamA) and a helicase (HamB) (7–9). Previous studies have suggested that HamB can serve as a gatekeeper, restraining HamA nuclease activity under non-infectious conditions and promoting HamA activation during phage infection or in response to DNA damage (7, 8). However, the nature of the activating input for HamAB remains unclear. Specifically, it is unknown whether DNA lesions, exposure of single-stranded DNA (ssDNA), repair-associated breaks and gaps, or other DNA states arising during infection or DNA damage serve as activating signals, and how these signals are transmitted to the HamAB complex to initiate activation.

To gain insight into the upstream activating signals, we focused on AbpAB (AbpA–AbpB), a Hachiman-family variant encoded by *Escherichia coli* (10, 11). AbpA is a HamA-related nuclease that carries an additional N-terminal region, and AbpB is a helicase-like partner required for defense. For AbpAB, several key observations about upstream inputs have been reported (11). First, ectopic expression of gp32, the T4 phage ssDNA-binding protein (SSB), activates AbpAB and induces host growth arrest. Second, AbpAB can be activated even in the absence of phage under conditions that perturb genome maintenance, including DNA damage and mutations in DNA repair pathways. Together, these observations suggest that AbpAB may respond not only to infection itself but also to DNA states arising from perturbations in replication and repair, such as increased ssDNA or replication- and repair-associated DNA breaks and gaps.

As gp32 binds ssDNA and AbpAB responds to DNA damage and replication stress, host factors that process ssDNA and DNA states generated during replication or repair are likely to contribute to AbpAB activity. Among such factors, DNA polymerase I (PolA) is a multifunctional enzyme that plays a central role in DNA replication and repair pathways (12, 13). PolA is a 928-amino-acid protein and uniquely harbors an N-terminal 5′–3′ exonuclease domain (residues 1–323) not found in other DNA polymerases, in addition to its 5′–3′ DNA polymerase domain (residues 324–517) and 3′–5′ proofreading exonuclease domain (residues 521–928) (14). During Okazaki fragment maturation, PolA participates in the removal of RNA primers through its 5′–3′ exonuclease activity and simultaneously fills the resulting gaps via its polymerase activity. Furthermore, PolA is essential for processing ssDNA gaps and single-strand breaks (nicks) generated during base excision repair, nucleotide excision repair, and replication-associated DNA damage. Through these combined activities, including nick translation and gap filling, PolA contributes to the restoration of intact double-stranded DNA. Given its role in processing ssDNA intermediates generated during replication and repair, PolA represents a plausible factor supporting AbpAB activity.

RNase HI is another candidate potentially involved in AbpAB activity, given its established roles in DNA replication and repair pathways. *E. coli* possesses two RNase H enzymes, RNase HI and RNase HII, which are encoded by the *rnhA* and *rnhB* genes, respectively. RNase HI hydrolyzes the RNA strand of RNA-DNA hybrids and contributes to replication-associated RNA primer removal and the control of RNA-DNA hybrid structures (15). Notably, RNase HI has been reported to interact with bacterial SSB (16, 17), suggesting that it functions at replication and repair sites enriched in SSB-coated ssDNA. In this context, RNase HI may be positioned at the sites of active DNA metabolism where ssDNA intermediates are exposed during replication and repair. In *rnhA* mutants, persistent RNA-DNA hybrids interfere with replication fork progression, leading to fork stalling and collapse, which in turn can promote the accumulation of ssDNA regions, DNA repair intermediates, and genome instability. Overall, these processes may modulate AbpAB activity.

In this study, we focused on PolA and RNase HI—two major enzymes involved in DNA replication and repair—and used genetic and biochemical approaches to examine their roles in the activity of AbpAB and HamAB.

## RESULTS

### PolA is required for AbpAB activity

**Fig 1:**
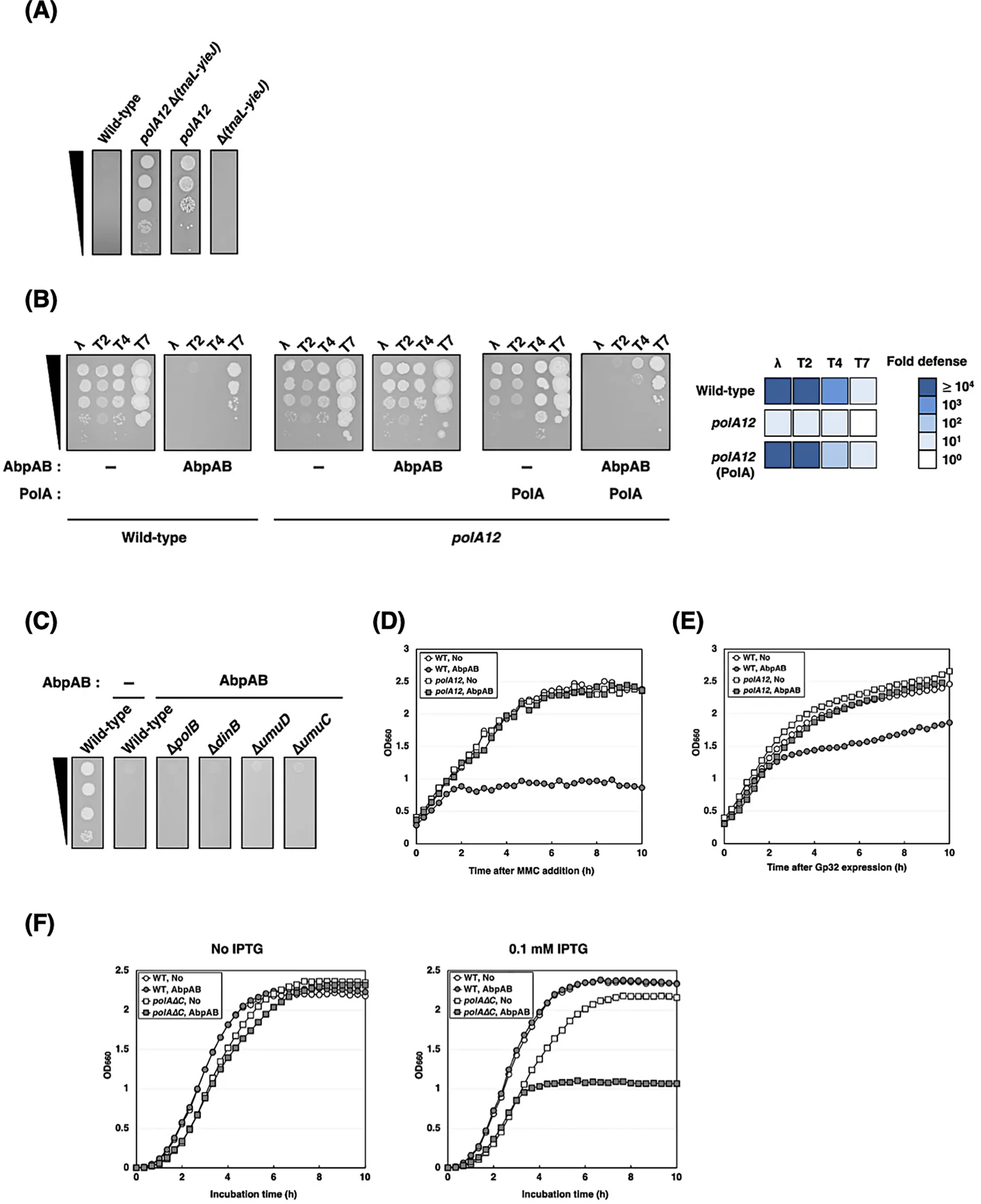
PolA-dependent AbpAB activity. (**A**) T4 phage propagation on *E. coli* mutants. In total, 10-fold serially diluted T4 phage suspensions were spotted onto LB agar plates seeded with ME5109 (wild-type), ME5001 (*polA12* ∆(*tnaL-yieJ*)), ME5000 (*polA12*), or BW25113∆(*tnaL-yieJ*) (∆(*tnaL-yieJ*)) harboring pST4-1 (constitutively expressing AbpA and AbpB) and incubated overnight at 30°C. (**B**) Phage propagation on the *polA* mutant strain. In total, 10-fold serially diluted phage suspensions (λ, T2, T4, or T7) were spotted onto LB agar plates containing 0.1 mM IPTG seeded with ME5109 (wild-type) or ME5000 (*polA12*) harboring pMW118 (–), pMW118-abpAB (AbpAB), pMW118 + pLG001 polA (PolA), or pMW118-abpAB + pLG001 polA and incubated overnight at 30°C. The protective effect (Fold defense) conferred by AbpAB in the wild-type, the *polA12* strain, or the *polA12* strain harboring pLG001-polA (PolA) was assessed using a serial dilution spot assay. Each experiment was performed with three biological replicates. (**C**) T4 phage propagation on the *polB*, *dinB*, *umuD*, or *umuC* mutant strain expressing AbpAB. In total, 10-fold serially diluted T4 phage suspensions were spotted onto LB agar plates seeded with BW25113 (wild-type), JW0059 (∆*polB*), JW0221 (∆*dinB*), JW1172 (∆*umuD*), or JW1173 (∆*umuC*) harboring either pBR322 (–) or pST4-1 (AbpAB) and incubated overnight at 30°C. (**D**) Growth of *E. coli* cells expressing AbpAB in the presence of MMC. ME5109 (WT) or ME5000 (*polA12*) harboring either pMW118 (No) or pMW118-abpAB (AbpAB) in LB broth containing 0.1 mM IPTG was treated with 1 µg/mL MMC when the OD_660_ reached approximately 0.4. (**E**) Growth of the *polA* mutant strain expressing AbpAB in the presence of T4 gp32. ME5109 (WT) or ME5000 (*polA12*) harboring pBAD33-gp32 plus pMW118 (No) or pMW118-abpAB (AbpAB) in LB broth containing 0.1 mM IPTG was treated with 0.2% L-ara when the OD_660_ reached approximately 0.4. (**F**) Growth of PolA C-terminal deletion mutant strain expressing AbpAB. ME5109 (WT) or ME9900 (*polA*∆*C*) harboring either pMW118 (No) or pMW118-abpAB (AbpAB) was treated with or without 0.1 mM IPTG when the OD_660_ reached approximately 0.5.

We performed a genetic screen using the Large-Scale Chromosomal Deletion Mutant Collection (NBRP-*E. coli* at NIG) to identify host factors involved in AbpAB-mediated phage defense. One strain, ME5001, supported T4 phage growth in the presence of AbpAB (Fig. 1A). To identify the responsible mutation, we analyzed individual mutants carrying either *polA12* or Δ(*tnaL-yieJ*). Spot assays showed that the *polA12* mutation, but not the Δ(*tnaL-yieJ*) deletion, restored T4 phage propagation in AbpAB-expressing cells. Although *polA* is not strictly essential in *E. coli*, it is conditionally required. The ME5000 (*polA12*) mutant used in this study showed markedly reduced 5′–3′ exonuclease and polymerase activities even under permissive conditions (18, 19). Furthermore, the *polA12* mutation impairs the replication of plasmids containing the ColE1 origin, such as pBR322, leading to a reduced plasmid copy number (20). To avoid this issue, we utilized the plasmid pMW118, which carries the pSC101 origin of replication instead of the ColE1 origin (21).

We again assessed the anti-phage activity of AbpAB in ME5000 harboring pMW118-abpAB (Fig. 1B). In the wild-type strain ME5109 harboring pMW118-abpAB, the propagation of λ, T2, and T4 phages was inhibited, whereas the T7 phage showed a marked reduction in plaque size. In contrast, in ME5000 harboring pMW118-abpAB, all phages propagated with an efficiency of plating (EOP) and plaque sizes comparable to those observed in ME5000 harboring the control plasmid pMW118. When PolA was expressed from the plasmid pLG001-polA in ME5000 harboring pMW118-abpAB, phage growth was inhibited, as observed in ME5109 harboring pMW118-abpAB. These results indicate that reduced PolA activity compromises AbpAB-mediated phage defense. In *E. coli*, DNA polymerases II, IV, and V function as SOS-inducible backup polymerases: Pol II mediates a relatively accurate replication restart (22), whereas Pol IV performs an error-prone translesion synthesis across damaged DNA (23). Pol V, composed of the UmuC catalytic subunit and truncated UmuD, mediates translesion DNA synthesis during the SOS response (24). As T4 phage failed to propagate on Pol II-, Pol IV-, or PolV-deficient cells harboring the plasmid pST4-1, which constitutively expresses AbpAB, none of these polymerases appear to be involved in AbpAB-mediated defense against T4 phage (Fig. 1C).

Next, we investigated whether PolA is necessary for AbpAB-mediated inhibition of bacterial growth after DNA damage. Consistent with previous results (11), ME5109 harboring pMW118 exhibited normal growth after mitomycin C (MMC) treatment, whereas ME5109 harboring pMW118-abpAB showed growth arrest upon MMC exposure (Fig. 1D). In contrast, ME5000 harboring pMW118-abpAB continued to grow normally after MMC treatment, indicating that PolA is necessary for the AbpAB-mediated bacterial growth inhibition following DNA damage.

AbpAB-expressing cells undergo growth arrest upon T4 phage gp32 expression (11). Therefore, we investigated whether PolA is also required for this growth arrest (Fig. 1E). ME5109 harboring pMW118 and pBAD33-gp32 grew normally after the induction of gp32 expression by arabinose. In contrast, ME5109 harboring both pMW118-abpAB and pBAD33-gp32 showed growth arrest after gp32 induction. Meanwhile, ME5000 carrying pMW118-abpAB and pBAD33-gp32 continued to grow after gp32 induction. These results demonstrate the necessity of PolA for AbpAB-mediated bacterial growth inhibition induced by gp32.

Finally, we examined whether the 3′–5′ exonuclease domain and polymerase domain of PolA are necessary for AbpAB activity. The N-terminal 5′–3′ exonuclease domain of PolA alone is sufficient for cell viability (25), and cells harboring the *polA1* allele containing an amber mutation at codon 342 remain viable (26). To assess the growth-inhibitory effect of AbpAB, we introduced the plasmid pMW118-abpAB into the *polA*Δ*C* strain ME9900, which lacks the C-terminal 587 amino acids and expresses only the N-terminal 341 residues, similar to the *polA1* mutant (27). In the absence of IPTG, ME9900 carrying either pMW118 or pMW118-abpAB showed normal growth comparable with that of the wild-type strain (Fig. 1F). However, upon IPTG induction, AbpAB expression in ME9900 harboring pMW118-abpAB resulted in rapid growth arrest, even without MMC treatment or gp32 expression. These results suggest that the N-terminal 5′–3′ exonuclease domain may contribute to AbpAB activity. Moreover, the simultaneous absence of both the 3′–5′ exonuclease and polymerase domains appears to promote AbpAB activity, leading to pronounced bacterial growth inhibition.

### PolA associates with the AbpAB complex

**Fig 2:**
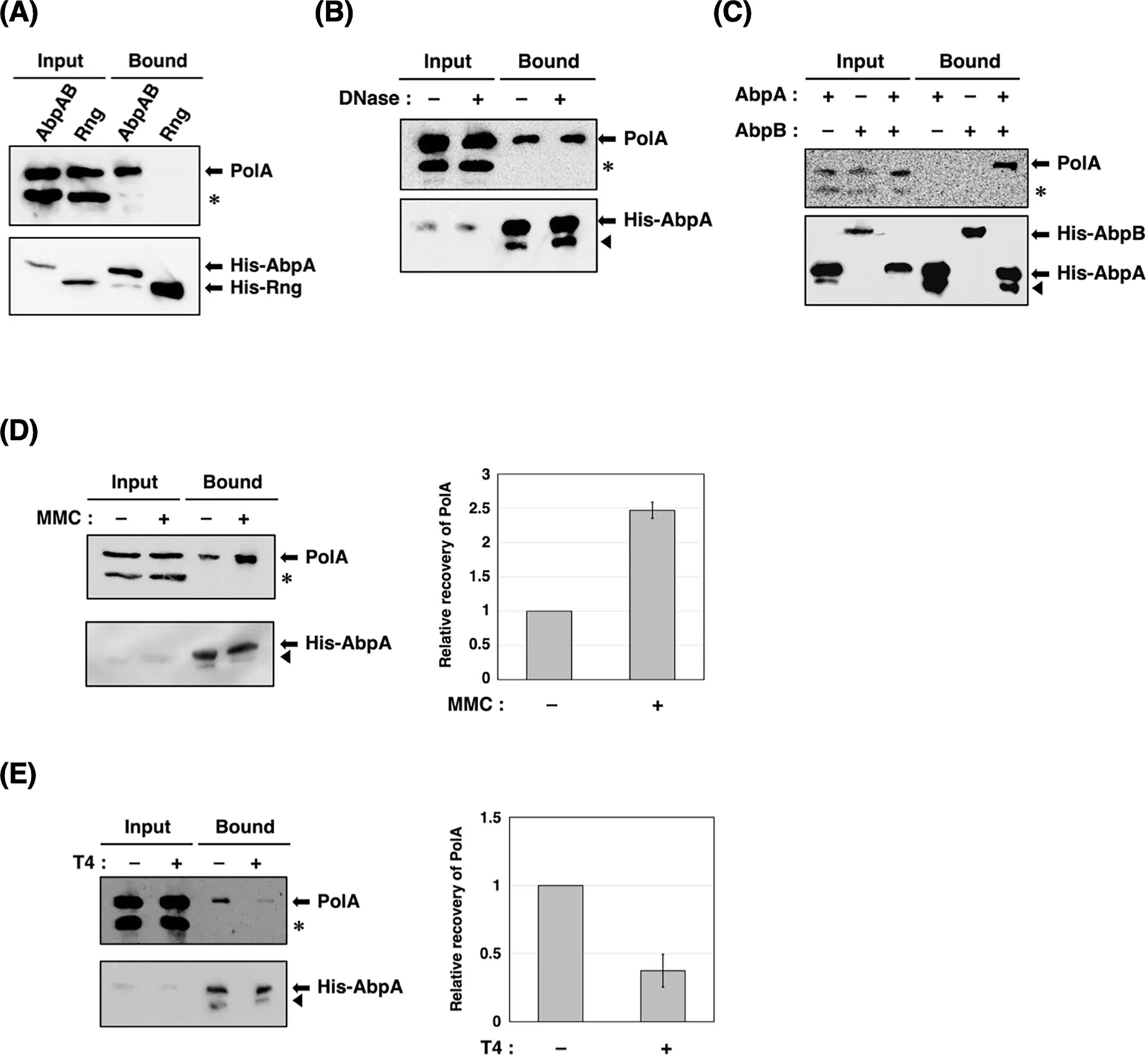
Association of PolA with the AbpAB complex. (**A**) Association between PolA and AbpAB. BW25113 cells harboring pQE80L-abpAB or pHU102 (expressing His-tagged Rng) were treated with MMC, and cell extracts were subjected to pull-down assays using Ni-NTA agarose beads. Input and bound proteins were analyzed using western blot with antibodies against PolA (upper panel) and His-tag (lower panel). The asterisk indicates a non-specific cross-reactive protein or a proteolytic fragment of PolA. (**B**) Association between PolA and AbpAB in the presence of DNase. BW25113 cells harboring pQE80L-abpAB were treated with MMC, cell extracts were treated with or without DNase and subjected to pull-down and western blot assays. The asterisk indicates a non-specific cross-reactive protein or a proteolytic fragment of PolA, whereas the arrowhead indicates a proteolytic fragment of His-tagged AbpA. (**C**) Association between PolA and AbpA or AbpB. BW25113 cells harboring pQE80L-abpAB, pQE80L-abpA, or pQE80L-abpB were treated with MMC. Cell extracts were subjected to pull-down and western blot assays. (**D and E**) Association between PolA and AbpAB following DNA damage induced by MMC or during T4 infection. BW25113 cells harboring pQE80L-abpAB were treated with or without MMC (**D**) or infected with or without T4 phage (**E**). Cell extracts were subjected to pull-down and western blot assays (left panels). Band intensities of PolA and His-tagged AbpA were quantified using ImageJ, and the amount of PolA was normalized to that of His-tagged AbpA. Quantification was based on three independent biological replicates (*n* = 3). For each experiment, the normalized PolA level in the control condition (–MMC or −T4) was set to 1, and the values for the treated samples (+MMC or +T4) are presented relative to the corresponding control (right graphs). Data are shown as mean ± SD (*n* = 3). The normalized +MMC and +T4 values were compared with one using a two-tailed one-sample *t*-test (*P* < 0.05).

To investigate the *in vivo* association between AbpAB and PolA, *E. coli* BW25113 cells were transformed with pQE80L-abpAB (expressing His-tagged AbpA and AbpB) or pHU102 (expressing His-tagged Rng as a negative control) and treated with MMC. Cell extracts were then subjected to pull-down assays using Ni-NTA agarose beads, and the eluted proteins were analyzed by western blotting. PolA co-eluted with His-AbpAB, but not with His-Rng, suggesting an association between AbpAB and PolA (Fig. 2A). Co-detection of PolA with AbpAB was independent of DNA, as PolA still co-eluted with His-AbpAB after DNase treatment (Fig. 2B). Furthermore, PolA did not co-elute with His-AbpA or His-AbpB alone, indicating that PolA associates with the AbpAB complex (Fig. 2C). Notably, MMC treatment significantly increased the amount of PolA recovered with His-AbpAB, suggesting that the association between PolA and AbpAB is enhanced following DNA damage (Fig. 2D). In contrast, 15 min after T4 infection, the amount of PolA recovered with His-AbpAB was significantly decreased, suggesting that the association between PolA and AbpAB is reduced during T4 infection (Fig. 2E).

### Loss of RNase HI induces AbpAB activity

**Fig 3:**
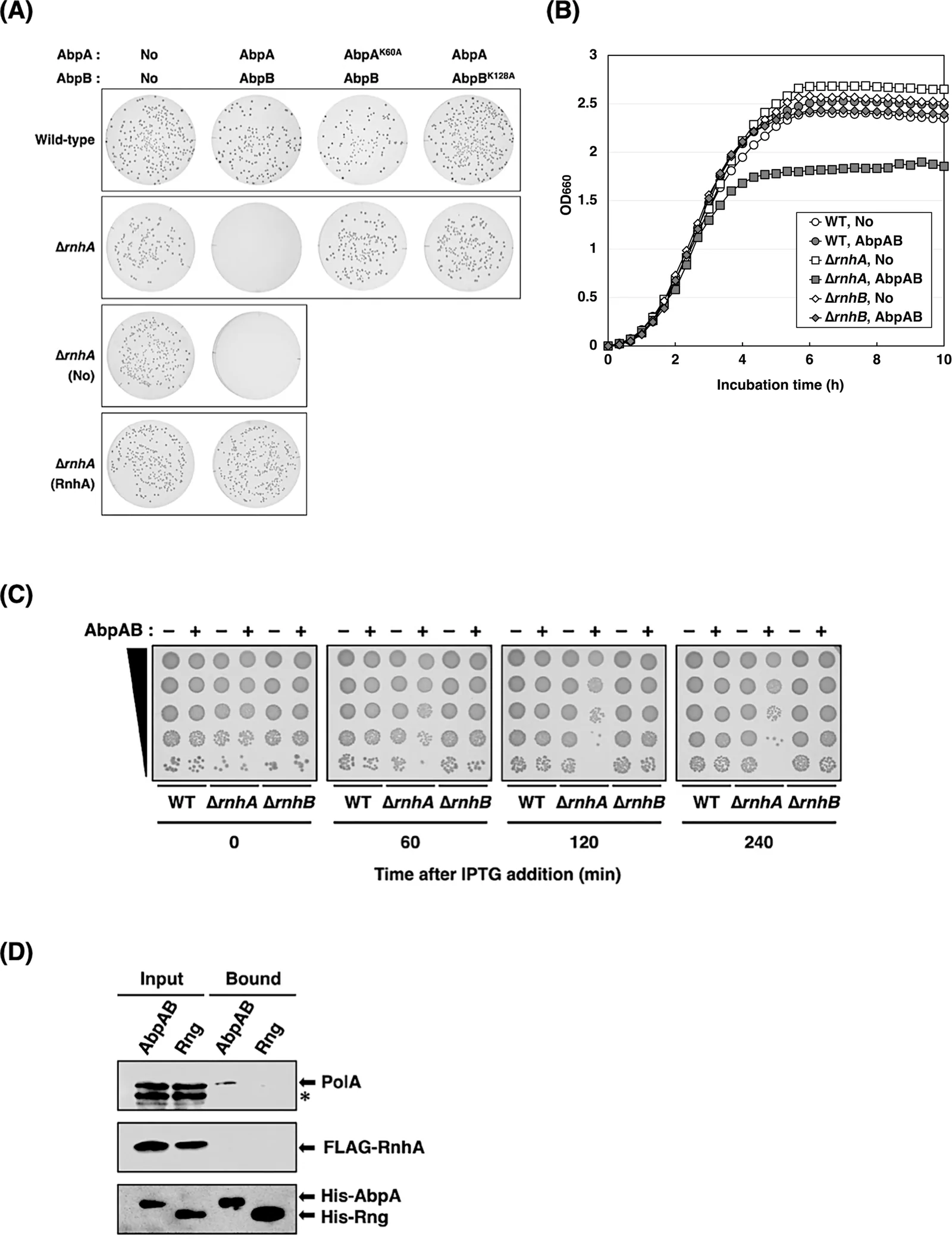
Induction of AbpAB activity by RNase HI loss. (**A**) Transformation of *E. coli* strains with various plasmids. Competent cells of BW25113 (wild-type), JW0204 (Δ*rnhA*), JW0204 harboring pLG001 (No), or JW0204 harboring pLG001-rnhA (RnhA) were transformed with 20 ng of pBR322 (No), pST4-1 (constitutively expressing AbpA and AbpB), pST4-14 (constitutively expressing AbpA^K60A^ and AbpB), or pST4-15 (constitutively expressing AbpA and AbpB^K128A^). Equal volumes of each transformation mixture were plated onto LB agar plates containing AMP and incubated overnight at 37°C. (**B**) *E. coli* cells expressing AbpAB. BW25113 (WT) , JW0204 (Δ*rnhA*), or JW0178 (∆*rnhB*) harboring either pMW118 (No) or pMW118-abpAB (AbpAB) were grown at 37°C. When the OD_660_ reached approximately 0.3–0.4, 0.1 mM IPTG was added. (**C**) The viability of *E. coli* cells expressing AbpAB. BW25113 (WT), JW0204 (Δ*rnhA*), or JW0178 (Δ*rnhB*) harboring either pMW118 (–) or pMW118-abpAB (+) was grown until the OD_660_ reached 0.3–0.4 and then treated with 0.1 mM IPTG for 0, 60, 120, and 240 min. Cell cultures were serially diluted 10-fold, and 3 µL of each dilution was spotted onto LB agar plates. The plates were incubated overnight at 37°C. (**D**) Association between RNase HI and AbpAB. Extracts from BW25113 cells harboring pBAD33-Flag-rnhA plus pQE80L-abpAB or pHU102 were subjected to pull-down assays using Ni-NTA agarose beads. Input and bound proteins were analyzed using western blot with antibodies against PolA (upper panel), FLAG-tag (middle panel), and His-tag (lower panel). The asterisk indicates a non-specific cross-reactive protein or a proteolytic fragment of PolA.

The plasmid pST4-1, which constitutively expresses AbpAB, was transformed into either a wild-type or an RNase HI-deficient strain (Δ*rnhA*) (Fig. 3A, Table 1). Transformants were successfully obtained from the wild-type cells, but none were recovered from Δ*rnhA* cells. When pST4-1 was transformed into Δ*rnhA* cells expressing RnhA from the plasmid pLG001-rnhA, transformants were obtained. To further investigate this phenomenon, we performed the same transformation experiments using plasmids pST4-14 or pST4-15, which harbor alanine substitutions at lysine 60 of AbpA or lysine 128 of AbpB, respectively, which are residues essential for anti-phage activity (11). Transformants were successfully obtained in Δ*rnhA* cells with both mutant plasmids. Therefore, the inability to obtain transformants with pST4-1 in Δ*rnhA* cells is likely attributed to AbpAB-mediated growth inhibition.

**TABLE 1:** Colony-forming units (CFU) in different strains^*a*^

Strain	Plasmid	Expression from plasmid	CFU/µg DNA
BW25113 (wild-type)	pBR322	**–** ^*b*^	1.6 × 10^4^
BW25113	pST4-1	AbpA and AbpB	2.2 × 10^4^
BW25113	pST4-14	AbpA^K60A^ and AbpB	4.7 × 10^4^
BW25113	pST4-15	AbpA and AbpB^K128A^	2.5 × 10^4^
BW25113	pBR322-hamAB	HamA and HamB	1.3 × 10^4^
JW0204 (∆*rnhA*)	pBR322	**–**	2.6 × 10^4^
JW0204	pST4-1	AbpA and AbpB	ND
JW0204	pST4-14	AbpA^K60A^ and AbpB	4.0 × 10^4^
JW0204	pST4-15	AbpA and AbpB^K128A^	4.2 × 10^4^
JW0204	pBR322-hamAB	HamA and HamB	2.0 × 10^4^
JW0204 with pLG001	pBR322	**–**	2.7 × 10^4^
JW0204 with pLG001	pST4-1	AbpA and AbpB	ND
JW0204 with pLG001	pBR322-hamAB	HamA and HamB	4.2 × 10^4^
JW0204 with pLG001-rnhA	pBR322	**–**	2.4 × 10^4^
JW0204 with pLG001-rnhA	pST4-1	AbpA and AbpB	3.6 × 10^4^
JW0204 with pLG001-rnhA	pBR322-hamAB	HamA and HamB	5.3 x 10^4^

^*a*^ Data are represented as the mean from three independent experiments. ND, not detected (<1 × 10^1^ CFU/µg DNA).

^*b*^ –, no protein expression.

To further investigate the role of RNase HI in AbpAB activity, we introduced pMW118-abpAB into the wild-type strain, the Δ*rnhA* strain, or an RNase HII-deficient strain (Δ*rnhB*). Transformants harboring either pMW118 or pMW118-abpAB were successfully obtained from all strains on LB agar plates lacking IPTG. These transformants were then cultured in liquid medium, and changes in growth following IPTG addition were monitored (Fig. 3B). Upon AbpAB induction, both wild-type and Δ*rnhB* cells continued to grow normally. In contrast, Δ*rnhA* cells exhibited marked growth retardation following AbpAB induction. Viable cell counts were measured in parallel (Fig. 3C). In contrast to Δ*rnhA* cells harboring pMW118, Δ*rnhA* cells carrying pMW118-abpAB showed a marked decrease in viable cell numbers, with cell viability decreasing to <1% within 2–4 h post-induction. These results demonstrate that loss of RNase HI induces AbpAB activity.

To investigate the *in vivo* association between RNase HI and AbpAB, extracts from cells expressing FLAG-RnhA and His-AbpAB were subjected to pull-down assays, and the eluted proteins were analyzed by western blotting. FLAG-RnhA did not co-elute with His-AbpAB, whereas PolA did, indicating no detectable association between RNase HI and AbpAB (Fig. 3D).

### HamAB activity requires PolA and is only marginally affected by RNase HI loss

**Fig 4:**
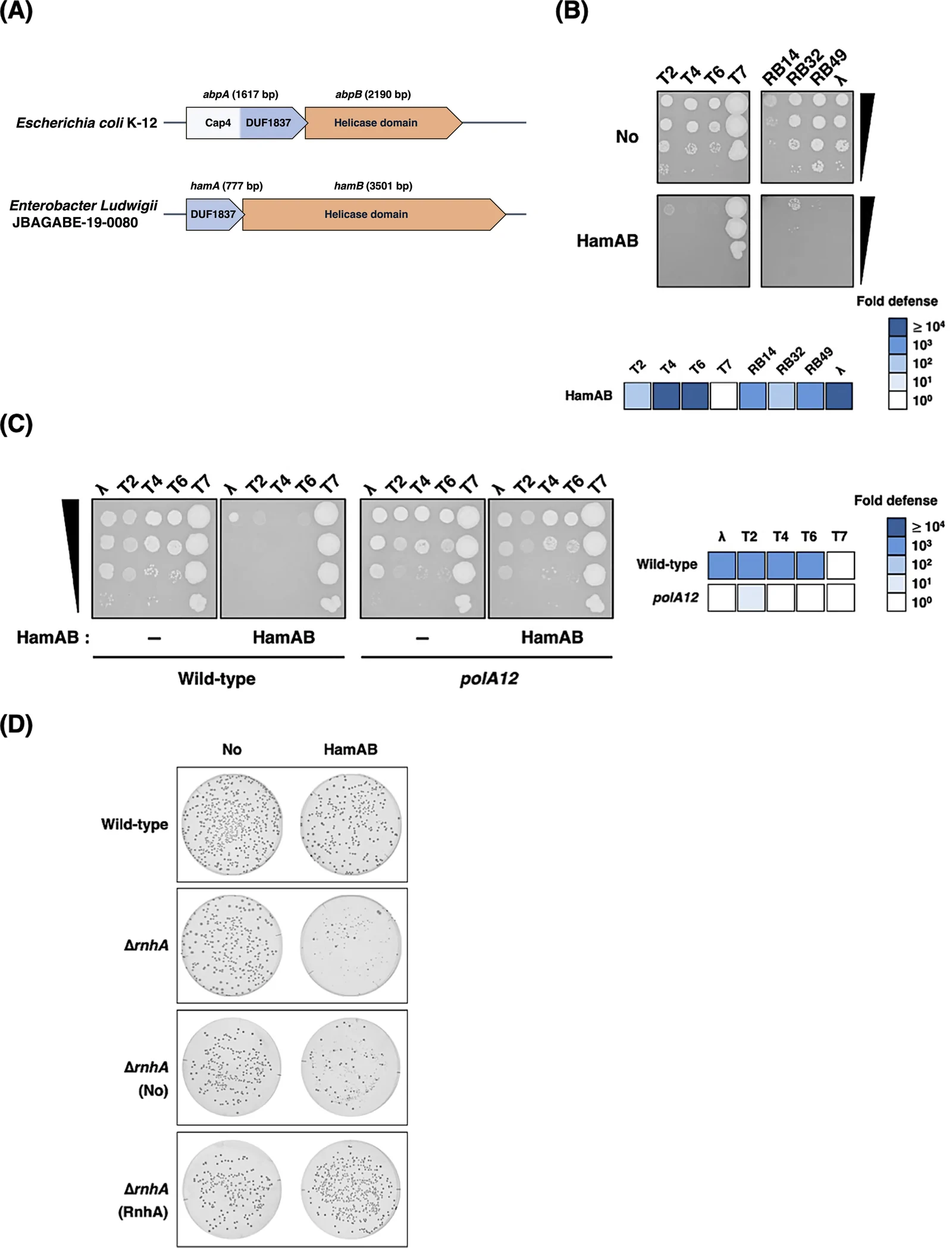
PolA-dependent HamAB activity with limited effects of RNase HI loss. (**A**) Comparison of the genetic organizations of AbpAB from *E. coli* K-12 and HamAB from the *E. ludwigii* isolate JBAGABE-19-0080. (**B**) Phage propagation on *E. coli* cells expressing HamAB. In total, 10-fold serially diluted phage suspensions (T2, T4, T6, T7, RB14, RB32, RB49, or λ) were spotted onto LB agar plates seeded with BW25113 harboring either pLG001 (No) or pKCN326 (constitutively expressing HamA and HamB) (HamAB) and incubated overnight at 30°C. The protective effect (Fold defense) conferred by HamAB was assessed using a serial dilution spot assay. Each experiment was performed with two biological replicates. (**C**) Phage propagation on the *polA* mutant strain. In total, 10-fold serially diluted phage suspensions (λ, T2, T4, T6, or T7) were spotted onto LB agar plates seeded with ME5109 (wild-type) or ME5000 (*polA12*) harboring either pLG001 (–) or pKCN326 (HamAB) and incubated overnight at 30°C. The protective effect (Fold defense) conferred by HamAB in the wild-type or the *polA12* strain was assessed using a serial dilution spot assay. Each experiment was performed with two biological replicates. (**D**) Transformation of *E. coli* strains with a plasmid expressing HamAB. Competent cells of BW25113 (wild-type), JW0204 (Δ*rnhA*), JW0204 harboring pLG001 (No), or JW0204 harboring pLG001-rnhA (RnhA) were transformed with 20 ng of pBR322 (No) or pBR322-hamAB (HamAB). Equal volumes of each transformation mixture were plated onto LB agar plates containing AMP and incubated overnight at 37°C.

We then investigated whether HamAB activity depends on PolA and whether it is induced by RNase HI loss. We identified a putative *hamA-hamB* operon in the genome of the clinical *Enterobacter ludwigii* isolate JBAGABE-19-0080 (Fig. 4A). The gene cluster, including its native promoter region, was cloned into the vector pLG001 to generate the plasmid pKCN326. We then examined whether HamAB from JBAGABE-19-0080 inhibits phage propagation (Fig. 4B). Although the propagation of T7 phage was unaffected, the growth of T2, T4, T6, RB14, RB32, RB49, and λ phages was significantly suppressed. These results confirm that HamAB confers phage defense.

Next, we examined the anti-phage activity of HamAB in *polA12* cells (Fig. 4C). As pLG001 and pKCN326 carry the p15A origin rather than ColE1, the *polA12* mutation does not affect their plasmid copy number. ME5109 and ME5000 harboring either pLG001 or pKCN326 were used as indicator cells to evaluate phage propagation. In ME5109 harboring pKCN326, the propagation of λ, T2, T4, and T6 phages, but not T7, was markedly suppressed. In contrast, all phages propagated with a comparable EOP and plaque size in ME5000 harboring pKCN326. These results, consistent with those observed for AbpAB, indicate that reduced PolA activity abolishes the anti-phage function of HamAB.

To investigate whether HamAB activity is induced by RNase HI loss, we subcloned the *hamAB* operon from pKCN326 into pBR322 to generate pBR322-hamAB. This plasmid was then transformed into wild-type and Δ*rnhA* cells (Fig. 4D, Table 1). While transformants were readily obtained in wild-type cells, the number of transformants was almost unchanged in Δ*rnhA* cells; however, most of the colonies (approximately 80%) were markedly smaller. In contrast, in Δ*rnhA* cells expressing RnhA from pLG001-rnhA, no small colonies were observed, and colonies exhibited normal size. Therefore, deletion of RNase HI has only a minor effect on HamAB-mediated activity.

## DISCUSSION

In this study, we analyzed how the Hachiman-family Abi defense systems AbpAB and HamAB depend on host enzymes involved in DNA replication and repair. We observed that *E. coli* DNA polymerase I (PolA) is required for AbpAB- and HamAB-mediated phage restriction, whereas RNase HI prevents AbpAB-dependent growth inhibition during uninfected growth. As Abi outputs are growth arrest or cell death, maintaining these systems in an inactive state in the absence of phage is essential for host fitness.

PolA is required for AbpAB-dependent growth inhibition itself, as in the *polA12* mutant, AbpAB failed to cause growth arrest under MMC treatment or upon gp32 expression (Fig. 1D and E). In *polA*Δ*C* cells expressing only the N-terminal region, AbpAB induction still caused strong growth arrest even without MMC treatment or gp32 expression (Fig. 1F), indicating that the C-terminal polymerase and 3′–5′ exonuclease domains are not required for this phenotype and that the N-terminal 5′–3′ exonuclease domain may contribute to AbpAB activity. This is consistent with the observation that AbpAB activity is reduced in the *polA12* strain, in which 5′–3′ exonuclease activity is impaired (Fig. 1B). The 5′–3′ exonuclease domain removes RNA primers during Okazaki fragment maturation and can process nicked or gapped DNA intermediates generated during replication and repair. Therefore, expression of only this domain could influence the processing or accumulation of replication or repair intermediates. A simple interpretation of the *polA*Δ*C* phenotype is that loss of the PolA polymerase domain slows or limits gap-filling DNA synthesis after primer removal or during repair, increasing the abundance or lifetime of nicked or gapped DNA. If AbpAB is triggered by such DNA structures, AbpAB induction in *polA*Δ*C* cells could be sufficient to cause growth arrest even without MMC or gp32 (Fig. 1F). However, given the potential for the polAΔ*C* background to itself generate replication or repair stress, this model remains speculative. Direct measurements of DNA lesions or replication and repair intermediates in *polA*Δ*C* cells or in cells overexpressing the 5′–3′ exonuclease domain will be needed to test this model.

Consistent with the idea that PolA acts close to AbpAB in the pathway leading to growth arrest, PolA was detected in His-AbpAB pull-down eluates. Furthermore, PolA co-eluted with the AbpAB complex, but not with AbpA or AbpB alone (Fig. 2C). Whether these observations reflect direct PolA–AbpAB binding, and whether such an association is required for AbpAB-dependent growth arrest and phage restriction, will require mutants that specifically weaken PolA–AbpAB association. We performed complex structure prediction using AlphaFold3 with AbpAB and PolA, which suggested a potential interaction across multiple models, most frequently involving the N-terminal region of PolA and AbpB within the AbpAB complex. However, the overall score for the complex prediction (0.8 × ipTM + 0.2 × pTM) was below 0.5, indicating limited confidence in the predicted complex structure. Further *in vivo* mutational analyses will be required to validate these models. In addition, high-resolution structural studies, such as X-ray crystallography and cryo-electron microscopy, will be necessary to further elucidate the interaction between PolA and the AbpAB complex.

Induction of AbpAB in Δ*rnhA* cells caused strong growth inhibition and a marked loss of viability (Fig. 3B and C). These results indicate that RNase HI normally suppresses AbpAB-dependent toxicity during uninfected growth. RNase HI degrades the RNA strand of RNA-DNA hybrids and participates in RNA primer processing during replication (15). In *rnhA* mutants, persistent RNA-DNA hybrids increase replication-associated stress and interfere with replication fork progression, leading to fork stalling and collapse. This can result in the accumulation of ssDNA regions and other DNA intermediates that may trigger AbpAB-dependent growth inhibition even in the absence of phage infection. This interpretation is consistent with prior observations that AbpAB-dependent growth inhibition can occur in DNA repair mutants such as *recB* and *recC* (11). However, the DNA structure(s) that act as the proximal trigger in Δ*rnhA* cells remain to be identified.

**Fig 5:**
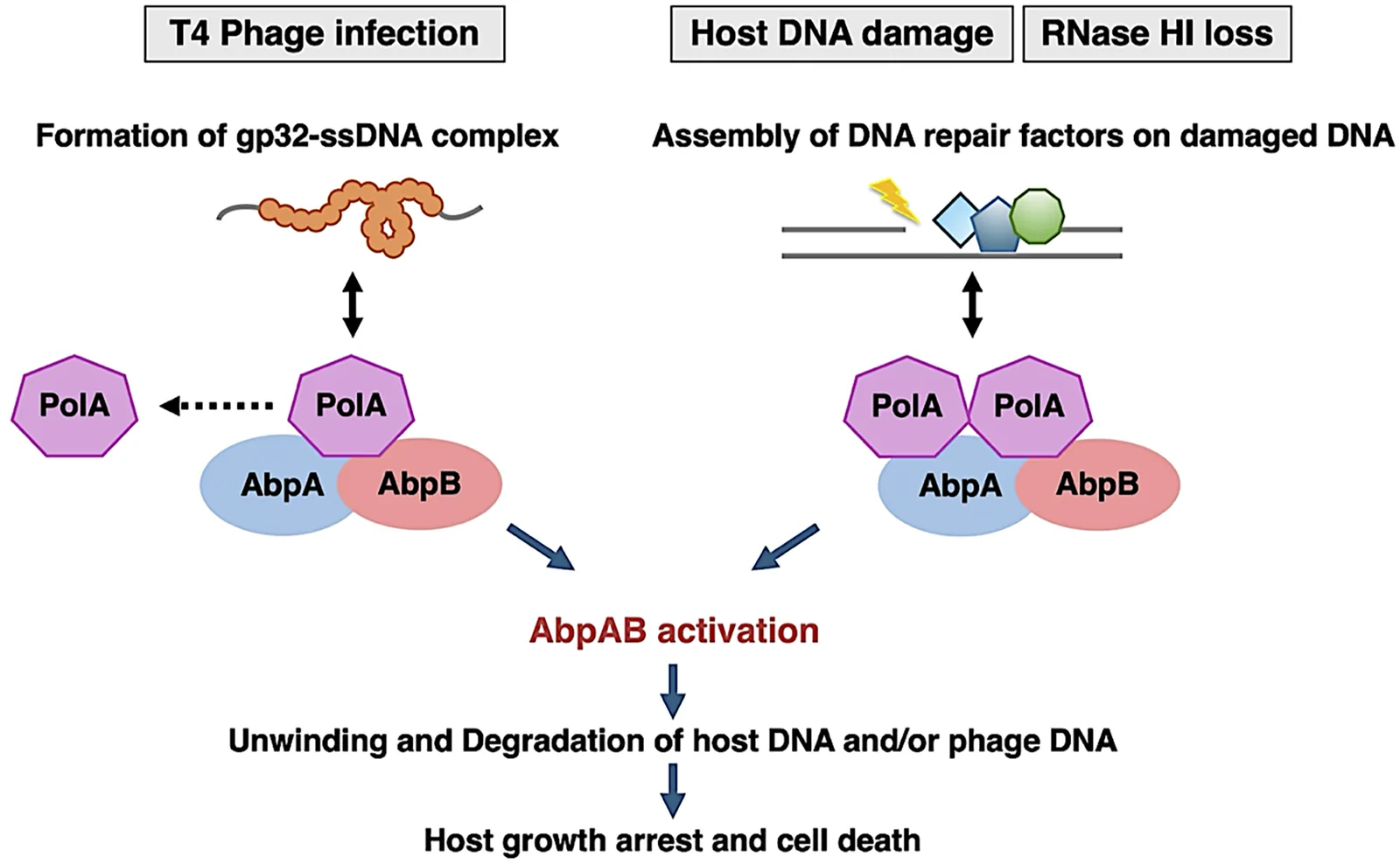
Proposed model for the regulation of PolA-mediated AbpAB activity. During phage infection, gp32–ssDNA complexes are formed. These complexes may associate with the PolA-AbpAB complex, resulting in reduced association between PolA and AbpAB. Alternatively, host DNA damage or loss of RNase HI may promote the recruitment of PolA-AbpAB complexes to damaged or aberrant DNA through the assembly of DNA repair factors. Although the underlying molecular mechanism remains unknown, both pathways are proposed to converge on AbpAB activation. Activated AbpAB may then promote the unwinding and degradation of host and/or phage DNA, resulting in host growth arrest and cell death, thereby contributing to abortive infection.

It was observed that the association between PolA and AbpAB was dynamically regulated under different conditions. PolA association with AbpAB increased after MMC treatment (Fig. 2D) and decreased following T4 infection (Fig. 2E). The reduction in association after T4 infection suggests that PolA may dissociate from the AbpAB complex during phage infection. Together with our findings that PolA is required both for AbpAB-mediated phage defense (Fig. 1B) and for AbpAB-mediated growth inhibition induced by gp32 (Fig. 1E) and that gp32 binding to ssDNA is required for this growth inhibition (11), these observations raise the possibility that PolA facilitates the association of AbpAB with gp32-bound ssDNA during T4 infection and subsequently dissociates from AbpAB after this association has been established (Fig. 5). Although this model remains speculative, it provides a possible explanation for why PolA is required for AbpAB activity despite its reduced association with AbpAB after infection. The mechanism and significance of the increased PolA and AbpAB association after MMC treatment (Fig. 2D) remain unclear. Because PolA is a DNA replication and repair factor that is recruited to sites of DNA nicks or gaps, one possibility is that PolA is recruited to damaged or aberrant DNA generated under these conditions and that AbpAB is co-recruited through its association with PolA (Fig. 5). In this model, PolA may recruit AbpAB to DNA replication or repair intermediates, thereby promoting AbpAB activity. Further experiments examining whether PolA recruits AbpAB to replication or repair intermediates will be required to test this hypothesis.

In summary, we identified two host replication and repair enzymes that strongly shape Hachiman-family Abi phenotypes. Our findings demonstrate that PolA is essential for AbpAB activity during phage infection and under conditions of host DNA damage, leading us to propose a model in which PolA mediates AbpAB activation (Fig. 5). Future studies defining the DNA structure(s) and molecular interactions that connect PolA and RNase HI to AbpAB or HamAB will be important for understanding how these Abi systems are activated during phage infection while remaining inactive during normal growth.

## MATERIALS AND METHODS

### *E. coli* strains and phages

All *E. coli* strains used in this study are listed in Table 2. BW25113, JW0059, JW0178, JW0204, JW0221, JW1172, JW1173, MG1655, ME5000, ME5001, ME5109, ME9806, and ME9900 strains were provided by the National BioResource Project (National Institute of Genetics, Japan). BW25113 ∆(*tnaL-yieJ*)::*kan* was constructed by GT7-mediated transduction of a kanamycin-resistance cassette from ME5001 (28). Bacteriophages T2L, T4D, T6, T7, λ RS45 (bla’-lacZSC), RB14, RB32, and RB49 were obtained from laboratory stocks.

**TABLE 2:** *E. coli* strains used in this study

Strain	Genotype	Source/Reference
BW25113	*rrnB3* ∆*lacZ4787 hsdR514* ∆(*araBAD)567* ∆(*rhaBAD)568 rph-1*	NBRP-*E. coli* at NIG
JW0059	BW25113 ∆*polB*::*kan^r^*	NBRP-*E. coli* at NIG
JW0178	BW25113 ∆*rnhB*::*kan^r^*	NBRP-*E. coli* at NIG
JW0204	BW25113 ∆*rnhA*::*kan^r^*	NBRP-*E. coli* at NIG
JW0221	BW25113 ∆*dinB*::*kan^r^*	NBRP-*E. coli* at NIG
JW1172	BW25113 ∆*umuD*::*kan^r^*	NBRP-E. *coli* at NIG
JW1173	BW25113 ∆*umuC*::*kan^r^*	NBRP-*E. coli* at NIG
MG1655	λ^-^ *ilvG^-^ rfb-50 rph-1*	NBRP-*E. coli* at NIG
ME5000	MG1655 *rpsL polA12 Zih*::*Tn10*	NBRP-*E. coli* at NIG
ME5001	MG1655 *rpsL polA12 Zih*::*Tn10* ∆(*tnaL-yieJ*)::*kan*	NBRP-*E. coli* at NIG
ME5109	MG1655 *rpsL*	NBRP-*E. coli* at NIG
ME9806	MG1655 ∆*hsdR ∆endA* ∆*recA*	NBRP-*E. coli* at NIG (27)
ME9900	MG1655 ∆*hsdR*::*frt polA∆C*::*kan*	NBRP-*E. coli* at NIG (27)
∆(*tnaL-yieJ*)::*kan*	BW25113 ∆(*tnaL-yieJ*)::*kan*	This study

### Plasmid construction

The primers used in this study are listed in Table 3. The Hachiman defense gene pair *hamA-hamB* was identified in the genome of the *Enterobacter ludwigii* isolate JBAGABE-19-0080 from the National Institute of Infectious Diseases using DefenseFinder (https://defensefinder.mdmlab.fr). The *hamA-hamB* locus, including its native promoter, was PCR-amplified from JBAGABE-19-0080 genomic DNA using primers KCO-0960 and KCO-0961. The pLG001 backbone was linearized using primers KCO-0021 and KCO-0022. The PCR product and linearized backbone were assembled using NEBuilder HiFi DNA Assembly (New England Biolabs, Ipswich, MA, USA) to generate the plasmid pKCN326. The plasmids pLG001-polA and pLG001-rnhA were constructed using the same method. The *polA* and *rnhA* loci, including their native promoters, were PCR-amplified from *E. coli* K-12 BW25113 genomic DNA using primers YO-1453 and YO-1454, and YO-1459 and YO-1460, respectively. The linearized pLG001 backbone and PCR products were assembled using NEBuilder HiFi DNA Assembly. The iVEC cloning strategy (27) was used for creating the plasmids pMW118-abpAB and pBR322-hamAB. Briefly, an insert DNA fragment containing genes *abpA-abpB* or *hamA-hamB* was PCR-amplified from pST4-1 or pKCN326 using primers YO-1071 and YO-1072 or YO-1158 and YO-1159, respectively. A vector DNA fragment for pMW118 (NIPPON GENE, Tokyo, Japan) or the pBR322 plasmid was PCR-amplified using primers YO-1057 and YO-1070 or YO-907 and YO-908, respectively. Insert and vector fragments were then transformed into ME9806 cells and plated on Luria-Bertani (LB) agar plates containing ampicillin (AMP; Nacalai Tesque, Kyoto, Japan). To generate pQE80L-abpAB or pQE80L-abpA, a DNA fragment containing *abpA-abpB* or *abpA* was PCR-amplified using pST4-1 as a template, and the primers YO-470 and YO-470-1 or YO-470 and YO-802, respectively. The PCR fragment was digested using *Bam*HI (NIPPON GENE) and *Hin*d III (NIPPON GENE) and ligated into the corresponding sites on pQE80L (Qiagen, Hilden, Germany). The DNA sequences of the constructed plasmids were confirmed via Sanger sequencing.

**TABLE 3:** Oligonucleotide sequences used in this study

Primer name	Sequence (5′–3′)
YO-470	TAGGATCCGAGTCGAACGATTCAGGCGG
YO-470-1	TCAAGCTTTCAATGTCGTCTAATACCGAG
YO-802	CGAAGCTTTCATACGACCTCTCTCACCTTC
YO-907	ATCCCGCAAGAGGCCCGGCAG
YO-908	ATCGTCCATTCCGACAGCATC
YO-1057	GCTGTAACAAGTTGTCTCAGGTGTTCAAT
YO-1070	AGCTGT TTCCTGTGTGAAATTG
YO-1071	CTGAGACAACTTGTTACAGCGTAGACGACCTAGCCTACCG
YO-1072	CAATTTCACACAGGAAACAGCTATGGAGTCGAACGATTCAGG
YO-1158	TGCCGGGCCTCTTGCGGGATAGACCAAGTGGTACAGCGTTTC
YO-1159	ATGCTGTCGGAATGGACGATTAGCCGCTTATGTCTATTGC
YO-1453	ATGCTAGTTATTGCGACTGCATGAAAAACAAAGACAAAC
YO-1454	CATTAATTGCGTTGCGCACAGCTTATGTTGCTTACTTACG
YO-1459	ATGCTAGTTATTGCGGGACTATTCCTTTAACGCTAAATCC
YO-1460	TAATTGCGTTGCGCACCGATCGGGTTTGAGAAAACATGG
KCO-0021	CGCAATAACTAGCATAACCCCTTGG
KCO-0022	TGCGCAACGCAATTAATGTAAGTTA
KCO-0960	TAATTGCGTTGCGCAAGACCAAGTGGTACAGCGTTTCCCG
KCO-0961	ATGCTAGTTATTGCGATAAGTCCTGCTACGGACTAGGATGTCAA
	

### *E. coli* growth and the colony-forming units (CFU) assay

*E. coli* cells were grown in LB broth supplemented with AMP and/or chloramphenicol (CM; Nacalai Tesque) at 30°C or 37°C. When the optical density at 660 nm (OD_660_) reached 0.3–0.5, 0.1 mM isopropylthio-β-galactoside (IPTG; Nacalai Tesque) for pMW118, 0.2% L-arabinose (L-ara; FUJIFILM Wako Chemical, Osaka, Japan) for pBAD (29), or 1 µg/mL mitomycin C (MMC; FUJIFILM Wako Chemical) was added as required. The cell density was monitored every 20 min using a biophotorecorder (TVS062CA; Advantec, Tokyo, Japan). Measurements were performed at least in triplicate. Representative results are shown in each figure. In the CFU assays, *E. coli* cells were grown in LB broth supplemented with AMP at 37°C until the OD_660_ reached 0.3–0.4 and were harvested at 0, 60, 120, and 240 min after adding IPTG. The cells were diluted with phosphate-buffered saline (Nacalai Tesque), plated on LB agar plates, and incubated at 37°C overnight.

### Pull-down and western blotting analyses

*E. coli* BW25113 cells harboring pQE80L-abpAB, pQE80L-abpA, pQE80L-His-abpB (10), or pHU102 (encoding His-tagged Rng (RNase G)) (4) were grown in 15 mL of LB broth supplemented with 50 µg/mL AMP at 30°C. Expression of His-tagged proteins was induced using 0.02 or 0.1 mM IPTG until the OD_600_ reached approximately 0.5. Cells were then subjected to the indicated treatments: incubation with 1 µg/mL MMC for 30 min, infection with T4 phage at a multiplicity of infection (MOI) of 5 for 15 min, or no treatment as a control. For pull-down of RNase HI, BW25113 cells harboring pQE80L-abpAB or pHU102 together with pBAD33-Flag-rnhA (30) were grown in 15 mL of LB broth supplemented with 50 µg/mL AMP and 30 µg/mL CM at 30°C. When the OD_600_ reached approximately 0.3, expression of His-tagged proteins and FLAG-tagged RnhA was induced with 0.1 mM IPTG and 0.1% L-ara, respectively, and cells were incubated for 1 h. Cells were harvested and suspended in 1 mL of lysis buffer containing 10 mM Tris-HCl pH 7.5 (FUJIFILM Wako Chemicals), 30 mM KCl (FUJIFILM Wako Chemicals), 10 mM magnesium acetate (FUJIFILM Wako Chemicals), and 20 mM imidazole (FUJIFILM Wako Chemicals). Cell suspensions were lysed by sonication, and the resulting lysates were clarified by centrifugation at 20,400 × *g* for 20 min. The resulting supernatants were treated with or without five units of DNase (RT Grade; NIPPON GENE) and then incubated overnight at 4°C with 20 µL of Ni-NTA agarose beads (Qiagen) by end-over-end rotation. The agarose beads were washed five times with 1.5 mL of washing buffer containing 10 mM Tris-HCl pH 7.5, 100 mM KCl, 10 mM magnesium acetate, and 20 mM imidazole. Subsequently, 60 µL of sample loading buffer containing 25 mM Tris-HCl pH 6.8 (FUJIFILM Wako Chemicals), 2.5% glycerol (FUJIFILM Wako Chemicals), 1% sodium dodecyl sulfate (SDS; FUJIFILM Wako Chemicals), 0.05% bromophenol blue (FUJIFILM Wako Chemicals), and 0.5% β-mercaptoethanol (FUJIFILM Wako Chemicals) was added to the beads. Samples were boiled at 98°C for 3 min, and input and bound proteins were separated by electrophoresis on 8% or 15% SDS-polyacrylamide gel and electroblotted onto Immuno-Blot PVDF membrane (Bio-Rad, Hercules, CA, USA). The membranes were probed with a mouse anti-His monoclonal antibody (GE Healthcare, Chicago, IL, USA) or a mouse anti-FLAG M2 monoclonal antibody (Sigma-Aldrich, St. Louis, MO, USA), followed by horseradish peroxidase-conjugated sheep anti-mouse IgG (Cytiva, Marlborough, MA, USA), or a rabbit anti-*E*. *coli* DNA polymerase I antibody (BioAcademia, Osaka, Japan), followed by horseradish peroxidase-conjugated donkey anti-rabbit IgG (Cytiva). Chemiluminescence was detected using Chemi-Lumi One Ultra (Nacalai Tesque) and Amersham ECL Prime (Cytiva) as substrates with a WSE-6100H LuminoGraph I imaging system (ATTO, Tokyo, Japan). Experiments were performed in duplicate or triplicate.
